# Antiviral Effect of Piperine on Chikungunya Virus: *In Vitro* Evidence and *In Silico* Analysis of E1-E2 Binding

**DOI:** 10.1021/acsomega.5c02814

**Published:** 2025-08-06

**Authors:** João Augusto Pereira da Rocha, Renato Araújo da Costa, Elaine Cristina Medeiros da Rocha, Alencar Kolinski Machado, Djenifer Leticia Ulrich Bick, Solange Binotto Fagan, Ana Lucia Monteiro Wanzeller, Micael Douglas de Souza Gomes, João Lucas Lima Rodrigues, José de Arimatéia Rodrigues do Rego, Davi do Socorro Barros Brasil, Anderson H. Lima

**Affiliations:** † Laboratory of Modeling and Computational Chemistry, Federal Institute of Education, Science and Technology of Pará (IFPA) Campus Bragança, Bragança 68600-000, PA, Brazil; ‡ Graduate Program in Chemistry, Federal University of Pará (UFPA), Belém 66075-110, PA, Brazil; § Laboratório de Planejamento e Desenvolvimento de Fármacos, Instituto de Ciências Exatas e Naturais, Universidade Federal do Pará, Belém 66075-110, Pará, Brazil; ∥ Laboratory of Molecular Biology, Evolution and Microbiology, Federal Institute of Education, Science and Technology of Pará (IFPA) Campus Abaetetuba, Abaetetuba 68440-000, PA, Brazil; ⊥ Graduate Program in Nanosciences, Franciscan University, Santa Maria 97010032, RS, Brazil; # Laboratory of Biosolutions and Bioplastics of the Amazon, Graduate Program in Science and Environment, Institute of Exact and Natural Sciences, Federal University of Pará (UFPA), Belém 66075-110, PA, Brazil; ∇ Laboratório de Isolamento Viral- Seção de Arbovirologia e Febres Hemorrágicas, Instituto Evandro Chagas, Ananindeua 67093-000, PA, Brazil

## Abstract

Chikungunya virus (CHIKV) is an emerging arbovirus that causes acute and chronic disease with significant public health concerns. Although recent vaccines have been licensed, no specific antiviral treatments are currently available. Targeting viral entry remains a promising strategy, particularly by inhibiting the E1-E2 glycoprotein complex, which mediates host cell attachment and membrane fusion. Natural products such as piperine have demonstrated broad-spectrum bioactivity, including antiviral properties, making them attractive candidates for drug discovery. This study employed a multidisciplinary approach, combining *in vitro* assays and computational modeling to evaluate the antiviral potential of piperine against CHIKV. Cytotoxicity assays were conducted in VERO cells, followed by plaque reduction assays to assess piperine’s effects at different stages of CHIKV infection. In parallel, molecular docking, MD simulations, and MM/GBSA calculations revealed a stable and energetically favorable binding of piperine to the E1–E2 fusion pocket. The results demonstrated that piperine exerts a time-dependent antiviral effect, with the most pronounced inhibition observed when administered during or after infection, suggesting activity beyond the initial entry step. Docking studies revealed that piperine binds within the E1-E2 fusion pocket, forming stable interactions with key residues such as MET88, LEU16, and TYR15, which are functionally important for CHIKV fusion. MD simulations confirmed that piperine maintains stable interactions at this interface and subtly alters the conformational dynamics of the glycoprotein complex. Cytotoxicity analysis showed that piperine is safe at low concentrations (0.001–10 μg/mL), while higher doses (≥100 μg/mL) induced oxidative stress and DNA damage in a dose-dependent manner. Collectively, these findings highlight piperine as a promising antiviral candidate targeting the CHIKV E1-E2 glycoprotein complex. Future studies should focus on structure-based optimization, pharmacokinetics, and *in vivo* efficacy to support the development of piperine-based antiviral therapies.

## Graphical abstract

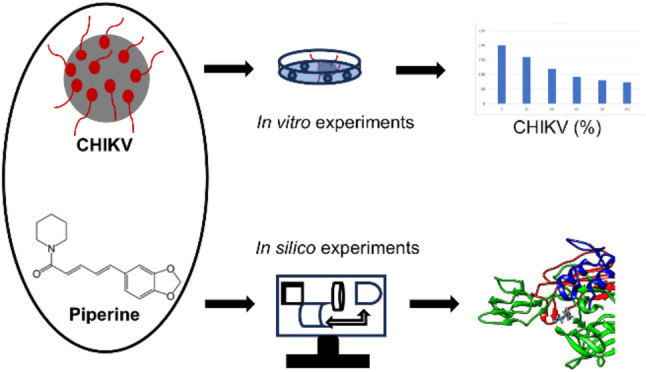

## 1 Introduction

Emerging and re-emerging viral infections represent a growing challenge to global public health, requiring the development of new antiviral therapies and vaccines to contain epidemic outbreaks. Among arboviruses, the Chikungunya virus (CHIKV) stands out due to its rapid spread and high morbidity, primarily affecting tropical and subtropical regions. Since its re-emergence in the 2000s, CHIKV has caused epidemic outbreaks associated with high fever, rash, and severe arthralgia, which can progress to debilitating chronic conditions that significantly impact patients’ quality of life.^1,2^ Although there are now two vaccines approved for Chikungunya prevention, specific antivirals remain unavailable, making the search for new therapeutic strategies essential.^3–6^

One effective approach in antiviral research is targeting viral entry mechanisms, as blocking this step can prevent infection and subsequent viral replication. Among the most promising therapeutic targets for CHIKV inhibition, the E1-E2 glycoprotein complex of the viral envelope stands out as a key player in the virus entry process into the host cell.^7,8^ The E2 protein mediates viral binding to the cellular receptor, while the E1 protein promotes the fusion of the viral membrane with the target cell, allowing the release of the viral genome into the cytoplasm.^9,10^ Thus, inhibiting the viral internalization process using bioactive molecules represents a strategic approach for developing new antivirals against CHIKV.^11,12^

The search for effective antivirals has prioritized compounds with high selectivity and low toxicity. In this regard, natural products have emerged as promising sources due to their structural diversity and historical success in drug discovery.^13–15^ For instance, flavonoids and alkaloids extracted from plants have demonstrated antiviral activity against SARS-CoV-2 and the Zika virus.^16^ In the context of arboviruses, compounds such as quercetin and curcumin have been investigated as potential CHIKV inhibitors, but new candidates are still needed.^17,18^

Piperine (1-piperoylpiperidine) is a widely studied alkaloid due to its various pharmacological activities, including antioxidant, anti-inflammatory, immunomodulatory, and antiviral effects.^19–26^ Clinical and preclinical studies have demonstrated that piperine modulates several cellular pathways, including the inhibition of nuclear factor kappa-B (NF-κB), Akt/mTOR, the NLRP3 inflammasome, cell cycle-related proteins, inflammatory cytokines, among others. Additionally, it is recognized for enhancing the bioavailability of other compounds by inhibiting drug-metabolizing enzymes and cellular transporters such as cytochrome P450 and P-glycoprotein (P-gp).^24^ Molecular docking and molecular dynamics studies have identified piperine’s ability to efficiently bind to viral proteins, including the Methyltransferase (MTase) of the Dengue virus and the interferon inhibitory domain VP35 of the Ebola virus, even showing better performance than commercial antivirals such as ribavirin.^25^ Recent experimental evidence further supports piperine’s antiviral activity against alphaviruses, particularly CHIKV.^26^

The identification of antiviral compounds can be accelerated using computational approaches such as molecular docking, molecular dynamics simulations, and binding free energy calculations.^15,27^ These tools allow the assessment of small molecule interactions with viral biomolecular targets, providing detailed insights into the stability and binding affinity of the protein–ligand complex.^28,29^ In this study, we investigated the antiviral potential of piperine, isolated and characterized in previous studies,^16,30^ against CHIKV through both experimental and computational approaches. Initially, *in vitro* assays were conducted to assess the cytotoxicity and antiviral activity of piperine in CHIKV-infected cell lines. *In vitro* assays revealed that piperine inhibits CHIKV infection most effectively during viral adsorption, followed by significant effects in pretreatment and postentry stages. These results suggest that piperine primarily blocks virus-cell interaction but may also interfere with later stages of the viral cycle. To elucidate the primary mechanism, we focused on piperine’s interaction with the E1-E2 glycoprotein complex through molecular docking and dynamics simulations, identifying key residues critical for its activity. These findings highlight piperine’s potential as a multiphase agent against CHIKV, with implications for the development of broad-spectrum antivirals.

## 2 Materials and Methods

### 2.1 Safety Profile Assessments

VERO cells (African green monkey kidney epithelial cells—Chlorocebus aethiops) were commercially obtained from the Rio de Janeiro Cell Bank (BCRJ) and cultured in Medium 199 supplemented with 10% fetal bovine serum (FBS) and 1% (v/v) antibiotics (penicillin/streptomycin). Upon reaching optimal confluence for the experiments, the cells were seeded into 96-well plates at a density of 2.5 × 10^5^ cells/mL and subsequently treated with a concentration–response curve of piperine (0.001–1000 μg/mL) for 24, 48, and 72 h. After the incubation periods, colorimetric, fluorometric, and microscopic assays were performed to evaluate the effects of the treatments on the cells.

### 2.2 Cell Viability and Proliferation Assessment

Following the respective treatments, both the cells used for the safety profile assessment were tested for viability and proliferation using the MTT assay (3-[4,5-dimethylthiazol-2-yl]-2,5-diphenyltetrazolium bromide), according to the method described by Denizot and Lang.^31^ MTT is a yellow reagent that is metabolized by mitochondrial enzymes in viable cells, converting it into formazan crystals, which exhibit a purple coloration. Absorbance was measured at 570 nm using a microplate reader.

### 2.3 Determination of Reactive Oxygen Species (ROS)

ROS levels were semiquantitatively measured using the 2′,7′-dichlorofluorescein diacetate (DCFH-DA) assay, following the methodology described by Costa et al.^32^ The DCFH-DA reagent is deacetylated by intracellular enzymes to form DCFH, which reacts with ROS, generating dichlorodihydrofluorescein (DCF), a fluorescent molecule. Fluorescence intensity was measured using a microplate reader at an excitation wavelength of 488 nm and an emission wavelength of 525 nm.

### 2.4 Indirect Measurement of Nitric Oxide (NO)

Nitric oxide levels were indirectly assessed using the Griess reagent assay, following the protocol of Choi et al.^33^ This assay is based on the detection of nitrate and nitrite, which are nitric oxide metabolites. Absorbance was measured at 540 nm using a microplate reader.

### 2.5 Measurement of Extracellular dsDNA Release

The levels of extracellular dsDNA released into the medium were quantified using the PicoGreen reagent, as described by Ahn et al.^34^ PicoGreen exhibits high affinity for dsDNA and intercalates between its strands, emitting fluorescence. Fluorescence intensity was measured using a microplate reader at an excitation wavelength of 480 nm and an emission wavelength of 520 nm.

### 2.6 Statistical Analysis

The *in vitro* results were initially tabulated using Microsoft Excel 365. The data were then converted into percentages relative to the negative control. Statistical analysis was performed using GraphPad Prism version 10.1.1, employing one-way ANOVA followed by Tukey’s post hoc test. Results with *p* < 0.05 were considered statistically significant.

### 2.7 Preparation of Piperine Stock Solution for Antiviral Test

The piperine used in this study was isolated and characterized in previous works by our group, which confirmed its identity and purity.^16,30^ The piperine stock solution was prepared by dissolving 2.5 mg of piperine in 1 mL of DMSO, resulting in a final concentration of 2500 μg/mL.

### 2.8 Dilution of Piperine Concentrations

Three dilutions of the piperine stock solution were prepared at different concentrations:

I. 0.05 μg/mL: 0.32 μL of the stock solution was diluted in 1599.68 μL of maintenance medium without fetal bovine serum (FBS).
II. 0.1 μg/mL: 0.64 μL of the stock solution was diluted in 1599.36 μL of maintenance medium without FBS.
III. 1 μg/mL: 6.4 μL of the stock solution was diluted in 1593.6 μL of maintenance medium without FBS.

### 2.9 Antiviral Treatment Procedure

After preparing the dilutions, 100 μL of each diluted solution was added to cells at different stages of the viral cycle:

I. Before the viral adsorption period.
II. During the viral adsorption period.
III. After the viral adsorption period.

The viral adsorption period was conducted for 1 h at 37 °C using 100 PFU of the virus. After each treatment, the cells were maintained under the respective incubation conditions described in the antiviral assay.

### 2.10 Biosafety Procedures

All procedures were conducted in compliance with the guidelines and criteria established by the International Biosafety Committee for handling infectious agents classified as risk group 2 (BRAZIL/MS, 2017).^35^ The materials used in this study were transported for sterilization and disposed of following the quality control guidelines of the Arbovirology and Hemorrhagic Fevers Section of the Evandro Chagas Institute (SEARB/IEC).

### 2.11 Cell Culture

The VERO cell line used in this study was kindly provided by the Viral Isolation Laboratory of SEARB/IEC. The techniques performed followed the adapted protocols of Macleod and Langdon et al.^36–38^

### 2.12 VERO Cell Maintenance

The cells were maintained in Medium 199 supplemented with glutamine (2 mM), sodium bicarbonate (3 mM), penicillin/streptomycin (10^4^ IU/mL), fungizone (2.5 mg/mL), and 10% FBS for cell growth, with FBS reduced to 5% for maintenance.

The cells were trypsinized every 5 days. After removing the culture medium and gently washing the cell monolayer twice with phosphate-buffered saline (PBS), trypsin-EDTA was added. Following cell detachment, growth medium 199 was added, and the cell suspension was distributed into new culture flasks. Fresh medium was replenished every 2 days. The culture flasks were maintained at 37 °C in a humidified atmosphere with 5% CO_2_.

### 2.13 Determination of Cell Concentration for Experimental Models

After cell monolayer detachment, a 200 μL aliquot was collected, mixed with 200 μL of Trypan Blue dye, and 10 μL of this dilution was used for cell counting in a Neubauer chamber. Five different cell concentrations (1 × 10^5^, 1.5 × 10^5^, 2 × 10^5^, 2.5 × 10^5^, 3 × 10^5^) were tested in 24-well plates, followed by cell cycle analysis to determine the most suitable concentration for the experimental models.

### 2.14 Cell Kinetics Analysis

To assess cell proliferation, 1 mL of cells was seeded into 24-well plates at a predetermined concentration and cultured for seven different time points (24, 48, 72, 96, 120, 144, and 168 h) in Medium 199 in duplicate. Cells were dissociated at each time point, and the concentration was determined using a Neubauer chamber with a 1:2 dilution in Trypan Blue.

### 2.15 Viral Sample

The viral sample used in this study was part of the SEARB biobank—Chikungunya virus, Asian genotype, strain BeH803609.

### 2.16 Cell Infection and Viral Stock Preparation

The viral stock was obtained by infecting VERO cell cultures. When cultures reached 80% confluence, the culture medium was removed, and infection with the viral inoculum was performed. After infection, the culture was incubated at 37 °C for 1 h for viral adsorption. Subsequently, the maintenance medium was added. The cytopathic effect (CPE) was monitored every 24 h using an Axiovert S100 inverted microscope (Zeiss).

After CPE identification, the supernatant was clarified by centrifugation, aliquoted into prelabeled sterile Eppendorf tubes, and stored at −80 °C for future use.

### 2.17 Viral Titration

The viral titration of stock solutions was performed according to Tauro et al.,^39^ with modifications. VERO cells were seeded in 24-well plates, and after 48 h, the medium was discarded, followed by the addition of 100 μL of serial viral inoculum dilutions (10^–1^ to 10^–12^). The plates were incubated for 1 h for viral adsorption with gentle agitation every 15 min. After incubation, 3% carboxymethylcellulose (CMC) in 10× concentrated DMEM was added, and the plates were incubated for 5 days. Subsequently, 1.5 mL of 10% paraformaldehyde (PFA) was added for 4 h, followed by washing with running water. The monolayer was stained with 1.5 mL of crystal violet for 30 min, washed, and dried before infectious titer calculation.

### 2.18 Indirect Immunofluorescence

The technique was performed according to Gubler et al. (1984),^40^ with modifications, to detect the presence of CHIKV in VERO cells. Uninfected VERO cells were used as negative controls. The cells were detached, placed on slides, and fixed at −20 °C in 100% acetone for 10 min. Then, 10 μL of immune ascitic fluid from Group B mice (antiflavivirus) was added to the slide and incubated for 40 min in a humid chamber at 37 °C. After incubation, the slides were washed with PBS and immersed in PBS for 10 min at room temperature (25 °C), followed by a quick wash with distilled water. Next, 10 μL of fluorescein-labeled antimouse immunoglobulin G antibody (antimouse FITC-Sigma) was added and incubated for 40 min at 37 °C. The slides were washed again with PBS, immersed in PBS for 10 min, and washed with distilled water. Finally, the slides were mounted with buffered glycerin and covered with coverslips for visualization using a fluorescence microscope (Axiophot, Zeiss).

### 2.19 Antiviral Activity

The antiviral assays were conducted using the plaque reduction assay described by Burleson et al.,^41^ using noncytotoxic concentrations of piperine.

### 2.20 Influence on Different Stages of Viral Infection

After 48 h of incubation and cell confluence, a 24-well plate was divided into three groups, each corresponding to a specific incubation period of the compound in relation to viral adsorption. The assay was conducted in three stages, the groups were evaluated before, during, and after viral infection to determine the effect of piperine on different stages of the viral cycle.

### 2.21 Molecular Docking

The molecular structure of piperine was obtained from the PubChem database (CID: 638024), while the structural model of the E1 and E2 glycoproteins of the Chikungunya virus was retrieved from the Protein Data Bank (PDB), using the PDB ID: 3N42. To avoid interferences in the docking analyses, cocrystallized water molecules and ions were removed. Docking simulations were performed using Molegro Virtual Docker (MVD), with the following parameters: Grid center based on the studies by Battini et al. and Thannickal et al.,^10,42^ which identified a critical pocket at the E1-E2 interface (*X* = −39.59; *Y* = −32.94; *Z* = −24.38). Grid resolution: 0.30 Å; Number of runs: 10; Population size: 50; Maximum iterations: 1500; Energy threshold: 100.0; Binding site radius: 15 Å (defined around the central coordinates of the grid center).

### 2.22 Molecular Dynamics

To accurately represent the electrostatic interactions of piperine during molecular dynamics simulations, quantum mechanics (QM) optimization was performed using the Gaussian 09 software.^43^ The optimization employed the B3LYP (Becke, 3-parameter, Lee–Yang–Parr)^44^ functional combined with the cc-pVDZ basis set, providing a balance between computational efficiency and accuracy for organic molecules. After optimization, Restrained Electrostatic Potential (RESP)^45^ charges were calculated to ensure accurate ligand parametrization in molecular dynamics simulations.

This molecular dynamics protocol was adapted from previous studies conducted by our research group.^46–51^ Molecular dynamics simulations were conducted using the AMBER package (AMBER22),^52^ following a well-established protocol. The general AMBER force field (GAFF) and the AMBER ff14SB were used to describe parameters for the ligand and protein, respectively.^53^ The tLEaP module was used to solvate the system in a cubic TIP3P^54,55^ water box with a 12 Å buffer, applying periodic boundary conditions. Counterions were added to neutralize the system’s total charge, and additional ions were incorporated to adjust the ionic strength to 0.15 M, reproducing physiological conditions. In addition to the protein-piperine complex simulation, an APO form simulation (protein without ligand) was also performed as a control, allowing a detailed comparison of the effects of piperine binding on the structural stability of the system.

All simulations were conducted at pH 5.5, determined using the PROPKA server,^56^ consistent with the approach taken by Battini et al.^10^ This pH was selected to mimic the acidic endosomal environment, a crucial step in the Chikungunya virus fusion process with the host cell. Considering that viral entry requires a pH-dependent conformational rearrangement of glycoproteins, simulating at pH 5.5 ensures that interactions relevant to the fusion process are accurately captured.

Subsequently, energy minimization was carried out in four sequential steps, beginning with solvent relaxation, keeping the protein fixed, followed by protein relaxation, allowing movement only of hydrogen atoms. The third step involved simultaneous minimization of protein and solvent hydrogen atoms, and finally, a general system minimization without constraints. The minimization protocol combined the Steepest Descent and Conjugate Gradient algorithms, automatically alternating between them to ensure efficient energy convergence. After minimization, the systems were gradually heated to 300 K over 1 ns under NVT (constant volume) conditions, with positional restraints of 5 kcal/mol·Å^2^ applied to the solute. Density equilibration was performed under NPT (constant pressure) conditions, using a force constant of 1 kcal/mol·Å^2^, followed by an unrestricted equilibration for 50 ns.

Temperature control was maintained at 300 K using the Langevin thermostat, with a collision frequency of 2 ps^–1^. To ensure structural stability, the SHAKE algorithm^57^ was applied to constrain all bonds involving hydrogen atoms. Long-range electrostatic interactions were handled using the Particle Mesh Ewald (PME) method, with a 10 Å cutoff for nonbonded interactions. The production phase consisted of a 300 ns MD simulation under NPT conditions, without positional restraints. During the simulation, structural parameters of both the protein-piperine complex and the APO form were monitored to assess their stability. Several structural analyses were performed to evaluate the system’s behavior over time, including Root Mean Square Deviation (RMSD), which was calculated for backbone and ligand atoms to assess the overall stability of the complex. Root Mean Square Fluctuation (RMSF) was computed for protein Cα atoms and key atoms of piperine, providing information on residue flexibility. Radius of Gyration (*R*_g_) was analyzed to monitor the compactness of the system throughout the simulation, while Solvent Accessible Surface Area (SASA) was calculated to evaluate changes in solvent exposure over time.

### 2.23 Free Energy

To elucidate the binding affinity of piperine with the E1-E2 complex of the Chikungunya virus, we performed binding free energy calculations using the MM/GBSA method,^58^ implemented in the AmberTools22 package.^52^ The theoretical framework underlying this approach has been extensively described in previous studies.^50^ The binding free energy estimation and its decomposition were conducted based on the last 10 ns of the molecular dynamics simulation trajectories.

## 3 Results

### 3.1 Cell Viability and Cytotoxicity of Piperine in VERO Cells

Cell viability assays with piperine extract in VERO cells revealed a dose-dependent and time-dependent cytotoxicity pattern. As shown in Figure 1, at low concentrations (0.001–10 μg/mL), piperine did not induce significant cell damage, maintaining cell viability above 90% compared to the control. However, at higher concentrations (100–1000 μg/mL), a sharp decline in cell viability was observed, with reductions of up to 50% after 24 h and less than 20% after 72 h of exposure. Intermediate concentrations (10–100 μg/mL) exhibited significant toxicity after 48 and 72 h, with cell viability reductions ranging from 30 to 60%, suggesting a cumulative and progressive effect of the compound.

**Figure 1:**
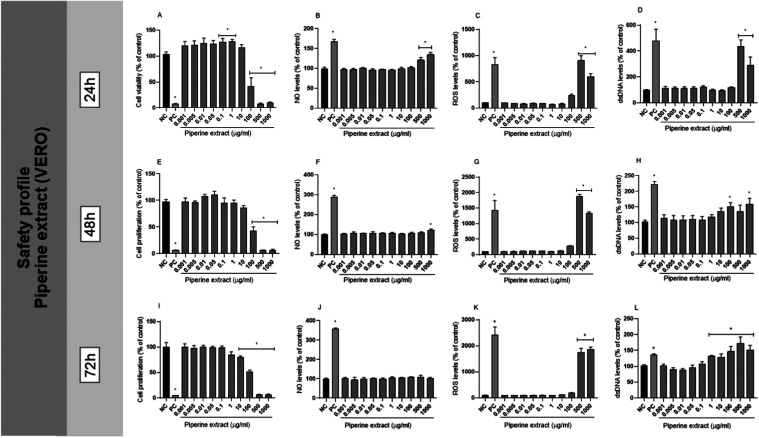
Safety profile of piperine in VERO cells. (A, E, I) Cell viability (24 h) and proliferation (48 and 72 h) (% of control) of exposure to different concentrations of piperine. (B, F, J) Nitric oxide (NO) levels (% of control) in VERO cells treated with piperine after 24, 48, and 72 h of incubation, respectively; (C, G, K) Reactive oxygen species (ROS) levels (% of control) at different piperine concentrations after 24, 48, and 72 h of incubation, respectively; (D, H, L) Quantification of extracellular double-stranded DNA (dsDNA) as an indicator of DNA damage after 24, 48, and 72 h of incubation, respectively. NC: negative control (cells under conventional cell culture condition). PC: cells exposed to 200 μM of H_2_O_2_ for MTT, DCFH-DA, and PicoGreen assays and 10 μM of sodium nitroprusside for NO determination assay. Values represent the mean ± standard deviation of at least three independent experiments. *p < 0.05, indicating a statistically significant difference compared to the negative control.

### 3.2 Time-Dependent Cytotoxicity Aspect

The exposure time to piperine significantly influenced toxicity. As demonstrated in Figure 1, at 24 h, cytotoxicity was less pronounced, even at intermediate concentrations (10–100 μg/mL). However, after 48 and 72 h, cell viability declined sharply at the same concentrations, indicating that piperine cytotoxicity intensifies over time. This effect may be related to oxidative stress accumulation and the activation of apoptotic pathways, as evidenced by the increased ROS and NO levels (Figure 1).

### 3.3 Reactive Oxygen Species (ROS) and Nitric Oxide (NO) Levels

ROS and NO levels were assessed to understand the mechanisms underlying piperine toxicity. At concentrations above 100 μg/mL, a significant increase in ROS and NO levels was observed (Figure 1), suggesting that oxidative stress may be one of the mechanisms involved in piperine’s cytotoxicity. This increase was more pronounced at 48 and 72 h, correlating with the reduction in cell viability. At lower concentrations (0.001–10 μg/mL), ROS and NO levels remained similar to the control, indicating that piperine does not induce oxidative stress under these conditions.

### 3.4 DNA Damage and Cell Proliferation

The quantification of extracellular double-stranded DNA (dsDNA), a marker of DNA damage, showed a significant increase at concentrations ≥100 μg/mL (*p* < 0.05) (Figure 1), especially after 48 and 72 h. This finding reinforces the hypothesis that piperine cytotoxicity may be associated with the induction of DNA lesions, possibly due to increased ROS and NO levels.

### 3.5 Statistical Analysis

Statistical analysis using one-way ANOVA, followed by Tukey’s post hoc test, confirmed that all differences between concentrations and exposure times were statistically significant (*p* < 0.05). Comparisons between 24, 48, and 72 h within the same concentrations also revealed relevant statistical differences, reinforcing the impact of exposure time on piperine toxicity (Figure 1).

### 3.6 Piperine’s Antiviral Activity Against CHIKV and Its Interaction with E1-E2 Glycoproteins

The antiviral potential of piperine against CHIKV was evaluated at two concentrations (0.5 μg/mL and 0.1 μg/mL), applied at three distinct stages of the viral replication cycle: before infection, during viral adsorption, and after infection. The relative viral load, normalized to the untreated viral control, is presented in Figure 2.

**Figure 2:**
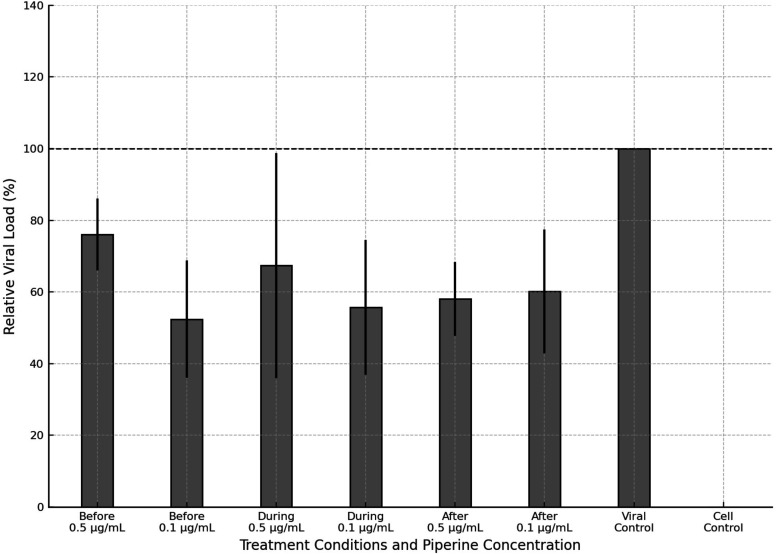
Antiviral effect of piperine at two concentrations (0.5 and 0.1 μg/mL) administered at different stages of the CHIKV infection cycle in VERO cells. The results represent the relative viral load (%) compared to the untreated viral control. Error bars denote the standard deviation from three independent experiments. The dashed line represents the viral control (100%).

The data obtained revealed a time-dependent antiviral effect of piperine. When administered during the viral adsorption phase, piperine reduced the relative viral load to approximately 67% at 0.5 μg/mL and 55% at 0.1 μg/mL, corresponding to reductions of about 33 and 45%, respectively, compared to the viral control. Similarly, postinfection treatment resulted in viral loads of approximately 58% (0.5 μg/mL) and 60% (0.1 μg/mL), representing reductions of 42 and 40%. In the prophylactic treatment (before infection), the viral load was reduced to ∼76% with 0.5 μg/mL (24% reduction) and ∼ 52% with 0.1 μg/mL (48% reduction). The viral control maintained 100% viral load, while no viral detection was observed in the cell control, validating the assay.

### 3.7 Molecular Docking

Molecular docking simulations between piperine and CHIKV E1-E2 glycoprotein complex revealed a significant affinity of the molecule for the fusion pocket, an important region for viral entry. The Moldock Score obtained was −138.8 in the Molegro Virtual Docker software. This dimensionless parameter evaluates the binding affinity of the compound to the molecular target, with more negative values indicating stronger interactions. Figure 3 presents a detailed interaction analysis, showing the formation of hydrogen bonds with residues His29 and Val229, as well as hydrophobic interactions with Met88, Leu16, Met70, and Thr175. These residues are located within the fusion pocket and directly contribute to the stable positioning of piperine at the E1-E2 complex interface.

**Figure 3:**
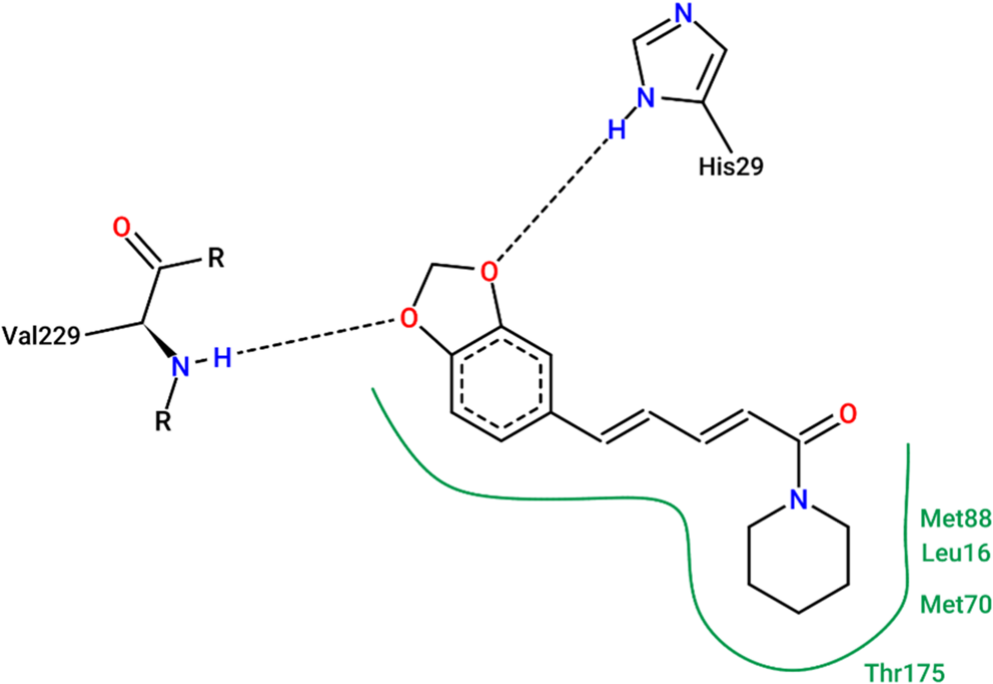
Molecular docking interactions between piperine and the CHIKV E1-E2 glycoprotein complex. Hydrogen bonds are observed with His29 and Val229, while hydrophobic interactions involve Met88, Leu16, Met70, and Thr175. The docking analysis suggests that piperine stabilizes the fusion pocket, potentially interfering with the structural rearrangement required for viral entry.

### 3.8 Molecular Dynamics

Molecular dynamics simulations were performed to assess the conformational stability of the CHIKV E1/E2 complex in the presence and absence of piperine. The parameters Root Mean Square Deviation (RMSD), Solvent Accessible Surface Area (SASA), and Radius of Gyration (Rg) were analyzed over a 300 ns trajectory, as they are fundamental for evaluating the structural stability of the complex. RMSD was calculated based on the protein backbone atoms (Cα, C, O, and N) to quantify structural variation over time. Figure 4 presents the analysis of the structural behavior of the systems.

**Figure 4:**
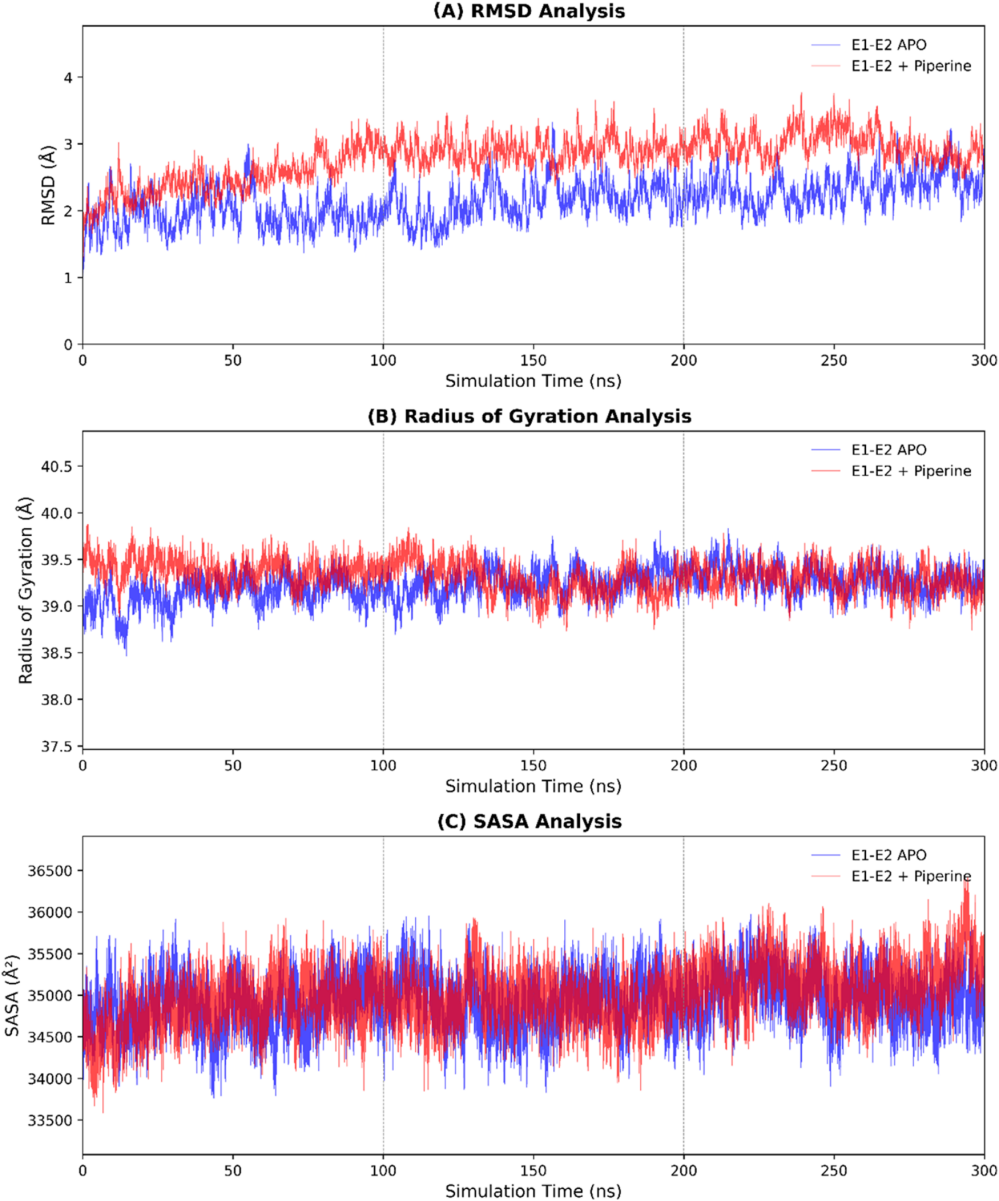
Structural stability analysis of the Chikungunya virus (CHIKV) E1/E2 complex in the presence and absence of piperine over a 300 ns molecular dynamics simulation. (A) Root Mean Square Deviation (RMSD), (B) Radius of Gyration (*R*_g_), and (C) Solvent Accessible Surface Area (SASA) are plotted as a function of time. The blue and red curves represent the complex bound to piperine and the apo form, respectively. These metrics provide insights into the conformational stability, compactness, and solvent exposure of the protein complex during the simulation.

The APO structure showed a mean RMSD of 2.14 ± 0.31 Å, whereas the Piperine–E1/E2 complex had a mean RMSD of 2.79 ± 0.33 Å. The trajectory indicates that the APO form stabilized more rapidly, while the piperine-bound system exhibited slightly greater fluctuations over the course of the simulation.

The SASA parameter was monitored to evaluate changes in the solvent-exposed surface area of the protein, and only minor variations were observed between the systems. Similarly, Rg was analyzed to assess the global compactness of the protein structure, with mean values of 39.23 ± 0.16 Å for the APO form and 39.32 ± 0.16 Å for the piperine-containing system.

To investigate the conformational impact of piperine binding to the CHIKV E1–E2 glycoprotein complex, we have performed Principal Component Analysis (PCA) using the Cα atoms from molecular dynamics simulations. The first two principal components (PC1 and PC2) were plotted to compare the dynamics of the APO and piperine-bound systems (Figure 5).

**Figure 5:**
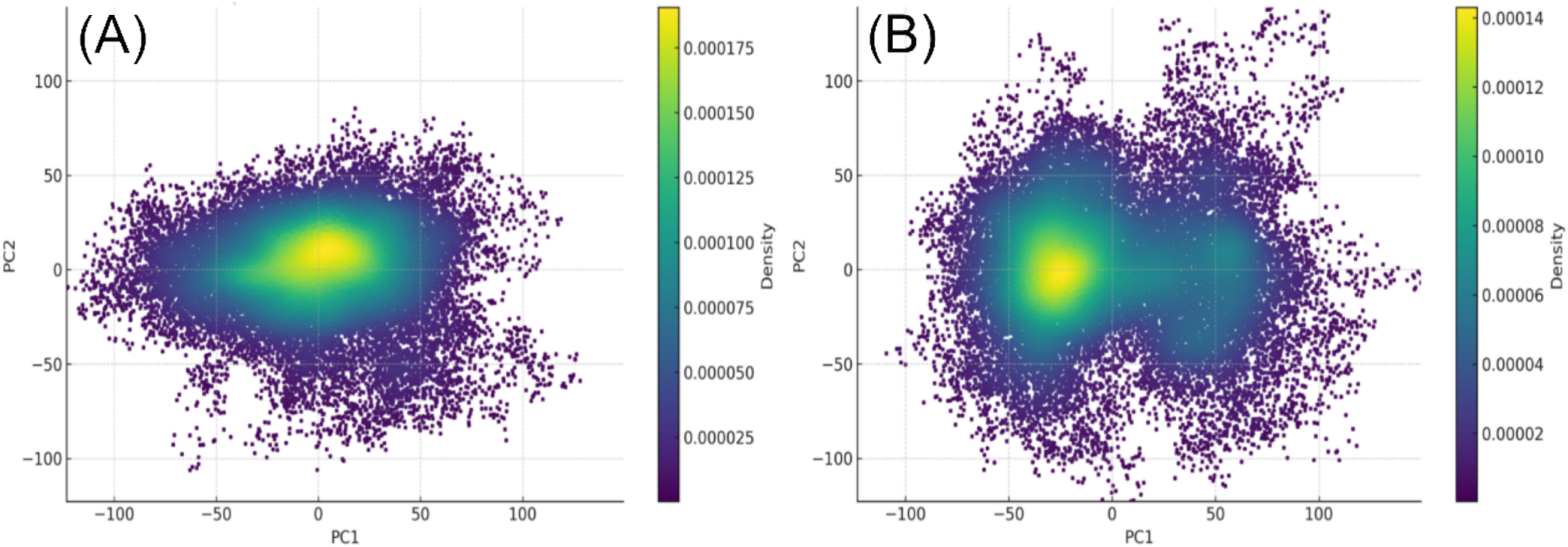
Principal Component Analysis of the CHIKV E1–E2 glycoprotein complex in the piperine-bound and APO states. The density distribution in the PC1–PC2 space indicates a more restricted conformational sampling in the piperine-bound system (A) compared to the broader and multimodal distribution observed in the APO system (B).

The PCA plot of the APO system (Figure 5B) revealed a broader and multimodal distribution, suggesting increased flexibility and sampling of diverse conformational subspaces in the absence of the ligand. In contrast, the piperine-bound system (Figure 5A) displayed a more compact and unimodal distribution, indicating restricted conformational mobility and stabilization of specific collective motions upon piperine binding. These results support the hypothesis that piperine stabilizes the E1–E2 complex by limiting its large-scale motions, potentially, those associated with membrane fusion. This ligand-induced reduction in essential dynamics may contribute to the observed inhibition of CHIKV entry and suggest a potential mechanism of antiviral action.

### 3.9 MMGBSA Calculations

The binding energy analysis between piperine and the E1-E2 glycoprotein complex of CHIKV was performed using the MM/GBSA method, considering the last 10 ns of molecular dynamics trajectories. The average values of binding free energy and its components are presented in Table 1.

**Table 1:** Binding Energies Obtained Using the MM/GBSA Method for the Interaction of Piperine with the CHIKV E1-E2 complex^a^

molecule	Δ*E*_vdW_	Δ*E*_ele_	Δ*G*_GB_	Δ*G*_nonpol_	Δ*G*_MM/GBSA_
Piperine	–53.0 ± 2.2	–13.9 ± 2.3	31.4 ± 1.7	–5.9 ± 0.1	–41.4 ± 3.6

a Values are presented as mean ± standard deviation (in kcal/mol). Δ*E*_vdW_ represents the van der Waals interaction contribution, Δ*E*_ele_ corresponds to the electrostatic contribution, Δ*G*_GB_ refers to polar solvation, Δ*G*_nonpol_ represents nonpolar solvation, and Δ*G*_MM/GBSA_ is the total free energy estimated as the sum of these components.

Per-residue energy decomposition analysis (Figure 6) was performed to identify the individual contribution of amino acids to the stability of the complex formed between piperine and the CHIKV E1/E2 glycoproteins. The residues showing the lowest interaction energies were LEU16 (−2.53 kcal/mol) and MET88 (−2.05 kcal/mol), followed by TRP89 (−1.64 kcal/mol), TYR15 (−1.65 kcal/mol), HIS18 (−1.63 kcal/mol), and VAL242 (−1.26 kcal/mol), all exhibiting favorable interactions with the ligand. For structural interpretation, residues were grouped based on their location within the viral complex subunits, being classified as part of the E1 glycoprotein (MET88, TRP89, and VAL229) or the E2 glycoprotein (TYR15, LEU16, ALA17, HIS18, MET70, and VAL242). The E1 region was predominantly associated with hydrophobic interactions, whereas the E2 region involved primarily van der Waals interactions and potential hydrogen bonds. Energetic analysis indicated that the greatest contribution to complex stabilization occurred within the E2 region, particularly involving residues LEU16 and TYR15, which presented the most favorable energetic values among all those analyzed.

**Figure 6:**
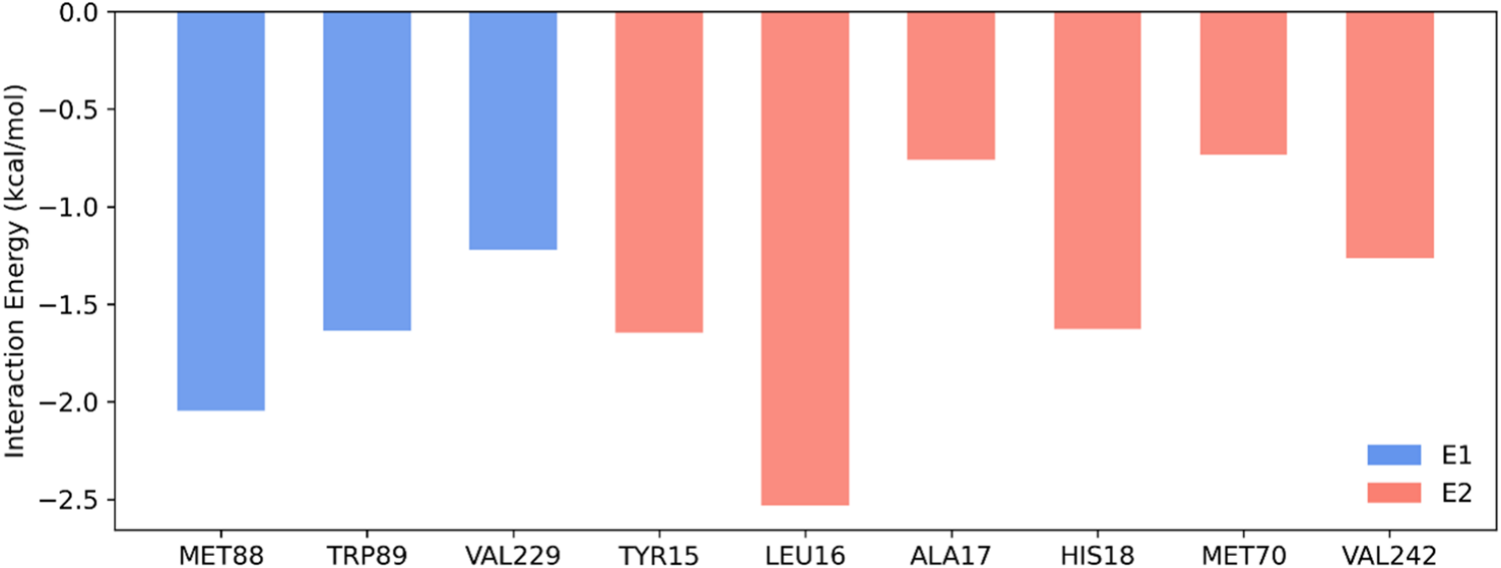
Residue decomposition energy analysis for the piperine-CHIKV E1-E2 glycoprotein complex. The bars represent the contribution of individual residues to the binding energy, categorized into two distinct regions: E1 (cyan) and E2 (orange). The most stabilizing residues were LEU16 (−2.53 kcal/mol) and MET88 (−2.05 kcal/mol), indicating their important role in complex stabilization. E1 residues primarily contributed through hydrophobic interactions, while E2 residues exhibited a mix of van der Waals forces and hydrogen bonding.

## 4 Discussion

The results of this study demonstrate that piperine exhibits a favorable safety profile at low concentrations, maintaining high cell viability and not inducing significant oxidative stress (Figure 1). However, at higher concentrations, piperine exhibits pronounced toxicity, with a drastic reduction in cell viability and increased ROS and NO levels. These findings are consistent with previous studies demonstrating the cytotoxicity of piperine in tumor cells, such as melanoma (B-16) and colon cancer (HCT-8).^59–61^ Similarly, Pareek et al. reported that piperine exhibits low cytotoxicity in nontumor cell lines such as BHK-21 and Vero.^26^

The time-dependent cytotoxicity is also a crucial factor. The increasing toxicity over time may be related to oxidative stress accumulation and the activation of apoptotic pathways, as suggested by the elevated ROS and NO levels. This behavior is frequently observed in natural bioactive compounds, particularly those that induce apoptosis through caspase activation.^62^ Although the study by Pareek et al. focused primarily on antiviral efficacy and acute cytotoxicity within 48 h, their results support the notion that piperine is safe at concentrations effective against viral replication. However, they did not explore oxidative stress markers or apoptosis pathways in detail, highlighting the importance of complementary analyses such as those performed here. Prolonged oxidative stress may trigger DNA damage and cell death mechanisms, as evidenced by the increase in extracellular dsDNA after 48 and 72 h.

The findings presented in Figure 2 indicate that piperine exhibits a more effective antiviral activity when administered prior CHIKV infection. At 0.1 μg/mL, viral load was reduced to approximately 52% in the preinfection group, while treatment with 0.5 μg/mL resulted in a ∼24% reduction. Although a formal selectivity index was not calculated in this study, the observed antiviral activity at noncytotoxic concentrations (≤10 μg/mL) supports the potential utility of piperine as a lead compound. Further studies are needed to establish dose–response profiles and accurately determine IC_50_ and selectivity index values, which will be essential for future preclinical evaluation. However, our findings suggest for the fisrt time that piperine interferes with early stages of viral infection, particularly viral entry. During the viral adsorption phase, both concentrations continued to demonstrate inhibitory effects, with viral loads remaining around 55–67% of the untreated control. This pattern reinforces the hypothesis that piperine may modulate the fusion dynamics or conformational flexibility of the E1-E2 interface at the moment of viral attachment and uptake.

Considering the role of the E1-E2 glycoprotein complex in CHIKV internalization, it is plausible that piperine interacts with these envelope proteins, impairing their function during the adsorption and fusion processes. E2 is responsible for binding to host cell receptors, while E1 mediates membrane fusion through a pH-dependent conformational rearrangement within endosomes. The antiviral activity observed during infection suggests that piperine may interfere with these early steps, disrupting viral attachment or fusion. This mechanism aligns with previous studies involving natural antivirals, such as polyphenols and alkaloids, which have been shown to inhibit E2-receptor binding interactions.^25^

The antiviral activity observed even when piperine was administered after viral infection suggests a potential action at later stages of the viral replication cycle, such as inhibition of viral RNA synthesis, interference with viral protein translation, or disruption of virion assembly.^63,64^ Notably, Pareek et al. demonstrated that piperine effectively inhibits viral replication by binding to the RNA-dependent RNA polymerase (nsP4), with low micromolar EC_50_ values and high affinity in SPR assays. These findings suggest that piperine may act as a multitarget antiviral, inhibiting both entry and replication processes in the CHIKV life cycle, and support further exploration of its therapeutic potential. This could expand the potential application of piperine not only as a prophylactic agent but also as a postinfection therapeutic intervention. Furthermore, piperine is known to possess anti-inflammatory, immunomodulatory, and antioxidant properties,^65^ which may contribute to reducing infection severity by limiting tissue damage and promoting a balanced immune response. These effects are particularly relevant during viral infections, as they directly influence disease progression and clinical outcomes.^66^

Previous studies have identified various natural compounds with inhibitory effects on CHIKV entry, including flavonoids, alkaloids, and terpenoids.^63–67^ Quercetin and curcumin, for instance, were shown to disrupt E2 binding by interacting with the viral envelope.^68–71^ These comparisons reinforce the idea that natural products can effectively target CHIKV entry mechanisms, and piperine represents a promising candidate within this category. Although initial studies demonstrate piperine’s promising antiviral activity against diverse viruses (including SARS-CoV-2, MERS-CoV, HCV, HBV, H1N1, and CHIKV), it should be noted that current evidence predominantly stems from in silico or *in vitro* analyses. As an example, Nag and Chowdhury^25^ employed molecular docking simulations to verify piperine’s inhibition of key enzymes in dengue and Ebola viruses. These findings, supported by other studies,^72–76^ indicate that piperine represents a therapeutic candidate for antiviral development, although its efficacy still requires validation through *in vivo* studies and clinical trials.

Our results demonstrate that piperine has the potential to inhibit CHIKV entry by directly binding to the functional pocket of the E1-E2 complex (Figure 3). Significantly, piperine interacts with key fusion residues (His29, Val229, Met88, and Met70) known to be essential for CHIKV membrane fusion. These findings corroborate the work of Battini et al.,^10^ which identified this region as a strategic target for blocking viral envelope-host cell membrane fusion. The similarity between the interactions observed for piperine and those described for previously reported inhibitors suggests that the compound may contribute to the stabilization of the closed conformation of the fusion pocket, thereby interfering with the structural rearrangement required not only for entry but also for subsequent events related to fusion and internalization. This hypothesis aligns with the antiviral effects observed during and after infection, which indicate a possible impact of piperine on stages following viral attachment. Additionally, the study by Thannickal et al.^42^ demonstrated that mutations in the E1 fusion region, such as E1-M88L and E1-N20Y, alter the conformation of E2, affecting its interaction with the Mxra8 receptor and cellular glycans such as heparin, further supporting the functional role of this interface in the viral entry process. If piperine successfully blocks this mechanism, it may act similarly to other antiviral compounds tested against alphaviruses, such as Arbidol, which inhibits viral fusion in enveloped viruses, and ribavirin, which has demonstrated antiviral effects against various RNA viruses, including alphaviruses.^77–84^

Another relevant aspect is that modulation of E1 conformation may reduce CHIKV’s dependence on cholesterol for membrane fusion, a phenomenon already described in association with specific mutations in the E1 region.^42^ Therefore, the effect of piperine on the structural arrangement of this pocket may help reduce viral infection by interfering with the fusion process, particularly during the early postattachment stages of the viral cycle. Supporting this hypothesis, molecular dynamics simulations revealed that the presence of piperine induces a slight increase in conformational deviation of the E1/E2 complex throughout the simulation (Figure 4), as evidenced by the higher average RMSD values compared to the APO structure. This behavior suggests a phibotential influence of the ligand on the conformational stability of the complex, possibly reflecting structural changes associated with its interaction within the fusion pocket. Despite the RMSD elevation, the SASA and radius of gyration (*R*_g_) values remained nearly constant between the two systems, indicating no significant changes in solvent exposure or overall protein compactness. These findings suggest that piperine promotes localized structural rearrangements without compromising the global integrity of the complex.

The per-residue energy decomposition analysis (Figure 6) revealed key interactions that contribute to the stability of the protein–ligand complex. The strong energetic contribution of LEU16 (−2.53 kcal/mol) suggests that this residue may be involved in the conformational stabilization of the complex, possibly influencing the functional structure of the target protein. This finding is consistent with structural studies demonstrating the importance of hydrophobic residues in stabilizing protein–ligand interactions.^10,42^ Molecular dynamics analysis has also revealed key hydrogen bonding interactions stabilizing piperine within the viral glycoprotein complex. The most persistent interactions occurred with LEU16, forming two hydrogen bonds with average distances of 3.2–3.3 Å and angles of ∼151°, which is consistent with its major energetic contribution identified by MM/GBSA analysis.

Additional stabilization occurred through interactions with PRO86 (3.2 Å, 151°) and PRO173 (3.2 Å, 145°), demonstrating the participation of both E1 and E2 subunits in ligand binding. Notably, the most geometrically favorable interaction was observed between the carbonyl group of piperine and the LEU16 backbone (3.0 Å, 157°), exhibiting near-ideal hydrogen bond geometry. Hydrogen bond combined with strong van der Waals contacts accounts for piperine’s stable binding (estimated Δ*G* = −41.4 kcal/mol) and its ability to modulate glycoprotein dynamics without disrupting overall complex integrity. The MET88 residue, which exhibited significant interaction energy, has previously been identified as a critical site for the stability of the CHIKV viral envelope.^42^ The study suggests that mutations at this position may alter the global conformation of the protein, affecting the affinity of specific ligands. In the context of piperine, the interaction with MET88 may play a key role not only in modulating the viral entry process but also in disrupting conformational transitions that are essential for subsequent steps of infection.

The classification of residues into E1 and E2 regions revealed distinct patterns of energetic contribution. While E1 exhibited more uniform hydrophobic interactions, E2 displayed more dynamic interactions, including potential hydrogen bonds. In the context of inhibitor development, the obtained results emphasize the importance of considering interaction hotspots within the protein structure. Future studies may explore structural modifications to piperine to optimize its affinity for critical residues such as LEU16 and MET88, enhancing its antiviral efficacy.

Despite these promising findings, further studies are needed to refine the understanding of its mechanism of action. Functional validation through mutagenesis of residues such as MET88 and LEU16, as well as binding affinity assays using SPR or ITC, will be critical for confirming target engagement. In addition, exploring the antiviral spectrum of piperine against related arboviruses, optimizing its structure for greater potency and selectivity, and assessing its pharmacokinetics and *in vivo* efficacy are essential next steps. Nanoformulations may further enhance its therapeutic potential by improving solubility and bioavailability.

## 5 Conclusions

This study provides compelling experimental and computational evidence supporting the antiviral potential of piperine against CHIKV. Molecular docking and molecular dynamics simulations demonstrated that piperine interacts stably with the E1-E2 glycoprotein complex, particularly at key fusion pocket residues such as MET88 and LEU16. In *in vitro* assays, piperine exhibited a time-dependent inhibitory effect, significantly reducing CHIKV infection when administered before or during viral entry, reinforcing its potential as an entry inhibitor. Binding free energy calculations further confirmed its energetic stability within the viral glycoprotein interface. The cytotoxicity profile of piperine revealed that it is safe at low concentrations (0.001–10 μg/mL), but higher doses induce oxidative stress and DNA damage, emphasizing the need for further pharmacokinetic and safety evaluations. Taken together, these findings indicate that piperine represents a promising candidate for antiviral drug development. Future studies should explore its mechanism of action, structural modifications, and *in vivo* efficacy, paving the way for the development of piperine-based therapeutics against CHIKV and other arboviruses.
